# DOA Estimation Based on Weighted l_1_-norm Sparse Representation for Low SNR Scenarios

**DOI:** 10.3390/s21134614

**Published:** 2021-07-05

**Authors:** Ming Zuo, Shuguo Xie, Xian Zhang, Meiling Yang

**Affiliations:** School of Electronic and Information Engineering, Beihang University, Beijing 100191, China; zuoming@buaa.edu.cn (M.Z.); zhangxian_66@buaa.edu.cn (X.Z.); meiling.yang@buaa.edu.cn (M.Y.)

**Keywords:** direction of arrival (DOA) estimation, sparse representation, low signal to noise, weighted l_1_-norm

## Abstract

In this paper, a weighted l_1_-norm is proposed in a l_1_-norm-based singular value decomposition (L1-SVD) algorithm, which can suppress spurious peaks and improve accuracy of direction of arrival (DOA) estimation for the low signal-to-noise (SNR) scenarios. The weighted matrix is determined by optimizing the orthogonality of subspace, and the weighted l_1_-norm is used as the minimum objective function to increase the signal sparsity. Thereby, the weighted matrix makes the l_1_-norm approximate the original l_0_-norm. Simulated results of orthogonal frequency division multiplexing (OFDM) signal demonstrate that the proposed algorithm has s narrower main lobe and lower side lobe with the characteristics of fewer snapshots and low sensitivity of misestimated signals, which can improve the resolution and accuracy of DOA estimation. Specifically, the proposed method exhibits a better performance than other works for the low SNR scenarios. Outdoor experimental results of OFDM signals show that the proposed algorithm is superior to other methods with a narrower main lobe and lower side lobe, which can be used for DOA estimation of UAV and pseudo base station.

## 1. Introduction

In the field of electromagnetic environment detection, direction of arrival (DOA) estimation is one of the most important techniques. In recent decades, DOA estimation has critical applications in radar systems, sonar systems, wireless communication systems, and radio astronomy systems [1,2,3]. In order to obtain higher accuracy of DOA estimation, a large number of algorithms have been proposed, such as the multiple signal classification (MUSIC) algorithm [4], rotation invariant subspace (ESPRIT) algorithm [5], maximum likelihood estimation [6], etc. However, multiple array elements and numerous snapshots are required in these algorithms, which brings great challenges to their application.

In recent years, the compressed sensing theory has been proposed for DOA estimation [7,8]. Since the array model can be transformed into a sparse representation, researchers pay more attention to DOA estimation based on sparse reconstruction. The author of [9] proposed the focal underdetermined system solver (FOCUSS) algorithm and verified the effectiveness of the sparse representation, but they only discussed the situation of a single snapshot, and the DOA estimation accuracy is poor under low SNR. Therefore, the FOCUSS algorithm based on multiple measurement vectors model evolved [10]. The literature [11] has studied the relationship between the narrowband array model and the compressed sensing model and proved the rationality of the array signal processing based on the compressed sensing theory. The solution of sparse reconstruction is the most ideal with the l_0_-norm as the minimized objective function, which is a problem of non-deterministic polynomial hard (NP-hard). Therefore, researchers use the l_p_ (0 < *p* ≤ 1) norm to approximate [12]. Many algorithms such as FOCUSS use the l_p_ (0 < *p* < 1) norm as the objective function, which is optimized by an iterative approximation method. The computation would become complicated with the increase in snapshots [9,10,13,14] by using the iterative method, which would be troubled by local extrema during optimization.

The l_1_-norm is able to satisfy the sparsity constraint and eliminate the possibility of local convergence of the objective function, which is beneficial for the solution. Therefore, the l_1_-norm-based singular value decomposition (L1-SVD) algorithm [12] proposed by Malioutov et al. is a classical DOA estimation method. The authors of [15] present a covariance matrix sparse representation method for DOA estimation. These methods are effective in high signal-to-noise (SNR) scenarios. However, most of these algorithms use l_1_-norm instead of l_0_-norm to obtain an approximate result. When the value of the SNR becomes low, the sparsity of the solution would become worse, and more spurious peaks will appear in the spatial spectrum.

To address this problem, the literature [16] uses iterative weighted l_1_-norm constraint minimization to increase the recoverable sparsity threshold and improve the recovery accuracy in the noise case. The orthogonality weighting of the noise subspace and the signal subspace is proposed, which enhances the robustness of the L1-SVD algorithm and improves the resolution of DOA estimation [17,18]. The literature [19] proposes a weighted norm penalty function from the capon spectrum. These methods can improve accuracy of DOA estimation at low SNR to some extent. However, when SNR becomes lower, especially when the SNR is lower than −12 dB, these methods will become worse.

In this paper, the weighted matrix is determined by optimizing the orthogonality of subspace, and the optimized weighted l_1_-norm is used as the objective function to minimize the signal sparsity, thereby improving the accuracy of DOA estimation and suppressing spurious peaks at low SNR.

The remainder of this paper is as follows: Section 2 introduces system model and the proposed method. The results are presented in Section 3, and a discussion is given in Section 4. Finally, Section 5 concludes this work.

## 2. System Model and the Proposed Method

### 2.1. Sparse Representation of Narrowband Array Signal

Assume that P far-field narrowband signals impinge on a uniform array with M elements (P < M). The distance of the adjacent antenna elements is equal to half of the wavelength. Then, the data model of the received signal at a time t can be determined by [4]:

(1) $$x\left(t\right)=\sum_{p=1}^{P}a\left(\theta_{p}\right)s_{p}\left(t\right)+n\left(t\right)(t=1,2,\ldots T)$$

where a(θp)=[1,e−i2πdsinθpλ,…,e−i2π(M−1)dsinθPλ]T is the M × 1 steering vector, d is the distance between two adjacent antenna elements, λ is signal wavelength, $\theta_{p}$ is the incident angle, and T is the number of snapshots. Formula (1) can be represented as vector form:

(2) X(t)=A(θ)S(t)+N(t)

where $A\left(\theta \right)=\left[\begin{array}{cccc} a\left(\theta_{1}\right), & a\left(\theta_{2}\right), & \ldots , & a\left(\theta_{P}\right) \end{array}\right]$ is the M × P steering matrix, $S\left(t\right)={\left[\begin{array}{cccc} s_{1}\left(t\right), & s_{2}\left(t\right), & \ldots , & s_{P}\left(t\right) \end{array}\right]}^{T}$ is the P × T sparse signal matrix. The additive white Gaussian noise is $N\left(t\right)=\left[\begin{array}{cccc} N_{1}\left(t\right), & N_{2}\left(t\right), & \ldots , & N_{M}\left(t\right) \end{array}\right]$ with zero mean and σ_n_^2^ variance. For the convenience of description, Formula (2) can be simplified as follows:

(3) X=AS+N

If **X** can recover **S**, then the DOA estimation of the source can be determined according to the position of the non-zero line in **S**. Formula (3) can be considered as a l_0_-norm problem, but the optimization of the l_0_-norm is a NP-hard problem. We use l_1_-norm instead of l_0_-norm to attain the approximate, and l_1_-norm is usually approximated [12] as follows:

(4) $$\min{\left\|X-AS\right\|}_{F}^{2}+h{\left\|S^{l_{2}}\right\|}_{l_{1}}$$

where $S^{l_{2}}$ is a column vector consisting of the l_2_-norm of each row in **S**, namely $S^{l_{2}}=\left[\begin{array}{cccc} s_{1}^{l_{2}}, & s_{2}^{l_{2}}, & \ldots , & s_{P}^{l_{2}} \end{array}\right]$. h is the regularization parameter affected by noise, which is usually a small constant. ${\left\|X-AS\right\|}_{F}$ is the result of straightening matrix X−AS by column and calculating l_2_-norm, that is:

(5) $${\left\|X-AS\right\|}_{F}^{2}={\left\|vec\left(X-AS\right)\right\|}_{l_{2}}^{2}$$

When the amount of snapshot T is large, it will lead to excessive calculation. Therefore, the L1-SVD algorithm performs SVD on the received data to obtain the M × P dimension reduction matrix **X**_sv_. The SVD of **X** is:

(6) $$X=U\Lambda V^{H}$$

Then, the sparse model after dimensionality reduction is derived as:

(7) $$\begin{array}{ll} X_{sv} & =XVD_{P}=ASVD_{P}+NVD_{P}\\ & =AS_{sv}+N_{sv} \end{array}$$

$S_{sv}=SVD_{P}$, $N_{sv}=NVD_{P}$, $D_{P}={\left[\begin{array}{cc} I_{P} & o \end{array}\right]}^{H}$, $I_{P}$ is the P × P dimensional identity matrix and 0 is the P × (T-P) dimensional zero matrix. In order to reduce the calculation and achieve better sparsity, Formula (7) can be preprocessed. Xsvw=Rx−12Xsv, Aw=Rx−12A. **R**_x_ is the covariance matrix of the original signal vector **X**. At this time, Formula (7) can be transformed into

(8) $$\min{\left\|X_{svw}-A_{w}S_{sv}\right\|}_{F}^{2}+h{\left\|S^{l_{2}}\right\|}_{l_{1}}$$

Using second-order cone programming (SOCP) to solve Formula (8), we obtain the results:

(9) $$\begin{array}{l} \min p+hq\\ s.t.{\left\|X_{svw}-A_{w}S_{sv}\right\|}_{F}^{2}\leq p\\ {\left\|S^{l_{2}}\right\|}_{l_{1}}\leq q \end{array}$$

### 2.2. Weighted l_1_-norm Method

Since the l_0_-norm is replaced by l_1_-norm in the L1-SVD algorithm, it would be difficult to guarantee the sparsity, especially when the value of the SNR is low. The constraint under l_1_-norm is the solution with the smallest modulus value. Here, the sparse signal **S** has a large modulus corresponding to a large coefficient, and a small coefficient corresponds to a small modulus. Therefore, the sparse signal **S** can be weighted to improve the sparsity of the solution.

Then, we can construct the weight according to the idea of multiple signal classification (MUSIC) algorithm, which can be obtained by orthogonal noise subspace and signal steering vector, namely

(10) $$b^{H}\left(\theta_{i}\right)E_{n}=0,i=\begin{array}{cccc} 1, & 2, & \ldots , & N \end{array}$$

where $b\left(\theta_{i}\right)$ is the array steering vector, ${\left(\right)}^{H}$ is the conjugate transposition, $E_{n}$ is the noise subspace, and N is the number of angles in space [0°,180°] divided at equal intervals. Due to the existence of noise, the process of signal, etc., the actual value of Equation (10) is not equal to 0, but a very small number. In other words, the projection of the signal array steering vector in the noise subspace is small, while the projection of the noise array steering vector is larger in the noise subspace. Therefore, a peak can be obtained in the signal direction by squaring the amplitude of the projected result and taking the reciprocal.

When the SNR is low, the orthogonality of signal subspace and noise subspace will become worse, the peak value of spectrum estimation will decrease, and the weight estimation will be inaccurate, which will lead to poor sparsity. This paper proposes that the weight is the array steering vector projected into the noise subspace divided by the value projected into the signal subspace. The formula is:

(11) wi=‖EnHb(θi)‖2‖EsHb(θi)‖2

The weighted matrix is expressed as:

(12) $$W=diag\left\{w_{i}\right\}$$

**Figure 1:**
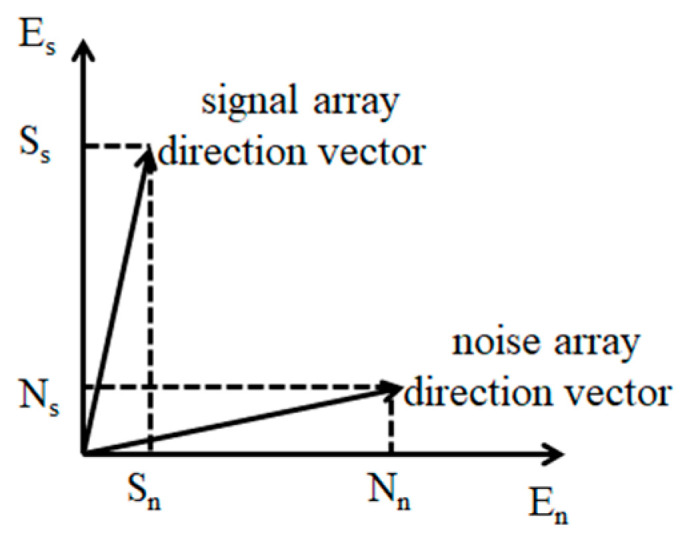
Projection analysis.

Figure 1 illustrates the projection of the signal array steering vector and noise array steering vector. The projection N_s_ of the noise array steering vector is relatively small in the signal subspace E_s_, while the projection N_n_ is relatively large in the noise subspace E_n_. On the contrary, the projection S_s_ of the signal array steering vector is large in the signal subspace E_s_, while the projection S_n_ is small in the noise subspace E_n_. When the algorithm is weighted by the literature [17], its peak ratio is N_n_/S_n_, while the proposed algorithm’s peak ratio is N_s_N_n_/S_s_S_n_ (N_s_/S_s_ < 1). Therefore, the weighted value decreases as the peak ratio decreases, and a small coefficient corresponds to a small modulus, which improves the sparsity of the solution. According to Formulas (8) and (12), we can obtain:

(13) $$\min{\left\|X_{svw}-A_{w}S_{sv}\right\|}_{F}^{2}+h{\left\|WS^{l_{2}}\right\|}_{l_{1}}$$

Convert to SOCP and solve

(14) $$\begin{array}{l} \min p+hq\\ s.t.{\left\|X_{svw}-A_{w}S_{sv}\right\|}_{F}^{2}\leq p\\ {\left\|WS^{l_{2}}\right\|}_{l_{1}}\leq q \end{array}$$

According to Formula (14), DOA can be estimated. The detailed steps of the algorithm are as follows:

(1) Decompose the data matrix to reduce dimension by the singular value, and preprocess **X***_sv_* and **A** to obtain **X***_svw_* and **A**_w_;
(2) Calculate the weight **W** according to Formula (12);
(3) Estimate the spectrum by using Formula (14).

## 3. Results

In this section, we study DOA estimation by using simulations and outdoor experiments of OFDM signals to verify the advantages of the proposed algorithm. A performance comparison of the proposed algorithm with other works, including the algorithms L1-SVD, W-L1-SVD [18], and C-L1-SVD [19], is presented and studied. We use a grid in the range of 0° to 180° with 1° spacing in this paper.

### 3.1. Simulation Results and Analysis

Three simulated examples are set out for the comparison. The simulation conditions are set as: (1) The simulated signal is based on orthogonal frequency division multiplexing (OFDM). As shown in Table 1, the parameters of the simulated OFDM are specified with a carrier frequency of 2038 MHz, a bandwidth of 20 MHz, 10 symbols, 256 subcarriers, 192 effective subcarriers, subcarrier spacing is 80 KHz, and the time of useful symbol length is 12.5 us. (2) The regularized parameter is *h* = 2.7. (3) Eight uniform arrays are used, in which the adjacent element distance is 0.06 m. (4) The number of snapshots is set as 3072.

#### 3.1.1. Simulation 1

**Figure 2:**
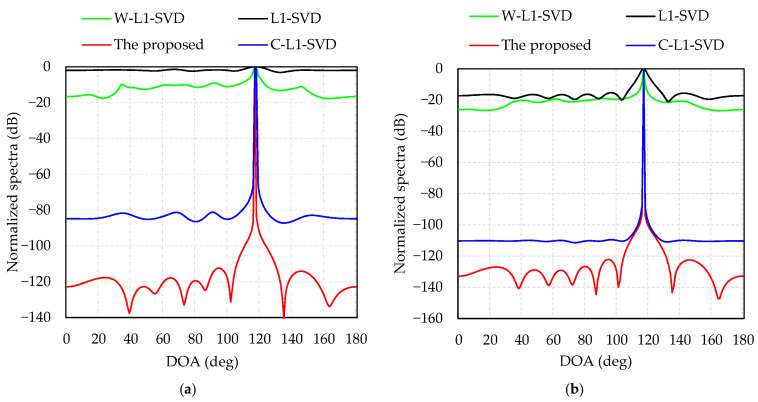
Normalized spectra of four algorithms: (**a**) SNR = −12 dB; (**b**) SNR = 0 dB.

Simulation 1 presents a performance comparison of the four algorithms at different levels of SNR. The direction of the incident wave is 117°. Figure 2a displays the normalized spectra of the four algorithms when SNR = −12 dB; as is shown, the DOA estimations of the proposed algorithm, L1-SVD, C-L1-SVD and W-L1-SVD are: 117°, 118°, 117°, and 117°, respectively. The L1-SVD algorithm shows the widest main lobe with a side lobe value of −2 dB, while the value is −16 dB for W-L1-SVD algorithm, but many spurious peaks exist. As for the C-L1-SVD algorithm, it is better than the former two algorithms with a side lobe value of −80 dB. In all these four algorithms, the proposed method shows the best performance with the lowest side lobes and sharpest main lobe. Figure 2b indicates the normalized spectra of four algorithms when SNR = 0 dB. The DOA estimations of the four algorithms are all 117°. In this case, although the spurious peaks of L1-SVD and W-L1-SVD decrease, the proposed method shows the best spectrum response with the lowest spurious peaks.

Comparing Figure 2a with Figure 2b, we can see that the spectra in L1-SVD, C-L1-SVD and W-L1-SVD have wide main lobes and high side lobes at a low SNR. When the SNR increases, the side lobes of the three algorithms will decrease, and their main lobes become sharper. As for the proposed algorithm, the main lobe is the sharpest and its side lobes are the lowest, which shows the most outstanding performance.

#### 3.1.2. Simulation 2

A comparison of root mean square error (RMSE) for the four algorithms is studied and presented in this case. The direction of the incident wave is 117°, and the SNR changes from −16 to 0 dB with a 2 dB step-size. For demonstration, 100 times Monte Carlo are operated for each SNR, and then the RMSE of DOA estimation be calculated as

(15) $$RMSE=\sqrt{\frac{1}{N_{c}P}\sum_{n_{c}=1}^{N_{c}}\sum_{p=1}^{P}{\left(\overset{\wedge }{\theta_{p}}\left(n_{c}\right)-\theta_{p}\right)}^{2},}$$

where *N*_c_ is the number of Monte Carlo simulations, $\theta_{p}$ is the real angle of signal, and $\overset{\wedge }{\theta_{p}}\left(n_{c}\right)$ is the DOA estimation of the *n*_c_ times Monte Carlo of the signal source.

**Figure 3:**
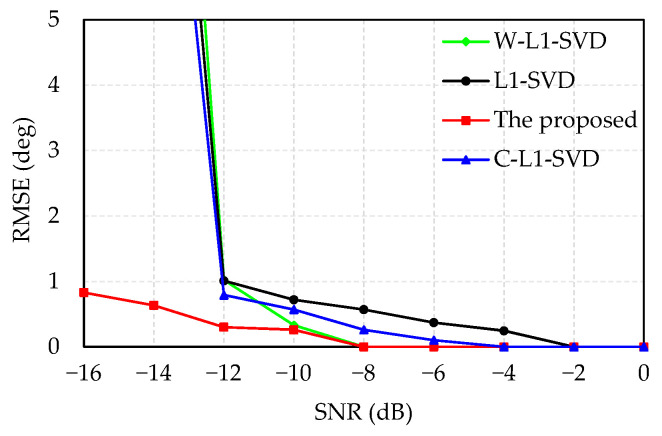
Relationship between SNR and RMSE.

In Figure 3, the simulated results demonstrate that RMSE decreases with the increased SNR. The proposed algorithm has better DOA estimation accuracy, which presents the lowest RMSE. When SNR is greater than −12 dB, the DOA estimation accuracy of the proposed algorithm is slightly higher than that of other three methods. When SNR is less than −12 dB, the DOA estimation accuracy of the other three algorithms drops sharply, while the estimation accuracy shows a minor decrease for the proposed algorithm.

#### 3.1.3. Simulation 3

Simulation 3 compares the resolution of the four algorithms at different SNRs. The simulation conditions are set as follows: (1) The two OFDM signals have the same power, carrier frequency and bandwidth. (2) The directions of the two incident waves are 80° and 100°, respectively.

**Figure 4:**
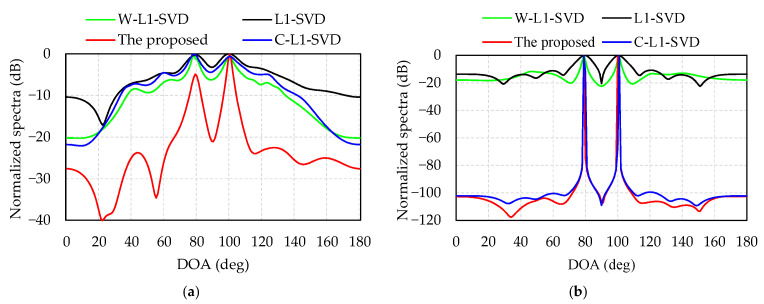
Resolution of four algorithms with two different SNRs: (**a**) SNR = –12 dB; (**b**) SNR = 0 dB.

Figure 4 provides the normalized spectra of the four algorithms with two different SNRs, −12 dB for Figure 4a and 0 dB for Figure 4b. Table 2 shows the DOA estimates of the four algorithms in Figure 4. All these four algorithms are able to distinguish the two DOAs in Table 2. It can also be observed that the side lobes decrease and their main lobes become sharper as the SNR increases. Additionally, the other three methods have higher side lobe with many spurious peaks, especially at low SNR, and the proposed algorithm has the sharpest main lobe and lowest side lobe.

The computer we run the Matlab program on is ThundeRobot Master N6, with a RAM of 16 GHz a main frequency of 3.2 GHz, and its CPU is i7-8700. Table 3 illustrates that, when SNR = −12 dB and the two incident angles are 80° and 100°, the accuracy and average calculation time over 100 times Monte Carlo of the four algorithms are compared. Shown in detail as Table 3, Since the L1-SVD and C-L1-SVD algorithms have many spurious peaks and low resolution at low SNR, they often result in DOA estimation errors. Therefore, the RMSE is significantly higher than W-L1-SVD and the proposed algorithms for two DOA estimations. It can be seen that since the four algorithms are all calculated by using SOCP, this would take up lots of calculation time, although the solution of the weighted value is different, resulting in similar running time for the four algorithms. Additionally, the proposed algorithm has lots of merits such as the smallest RMSE value, the highest accuracy and the best resolution. According to the results shown in Figure 4 and Table 3, although the operation time of the proposed algorithm is almost the same as the other three algorithms, its main lobe is the sharpest and the side lobe is the lowest, which exhibits a better resolution at low SNR.

#### 3.1.4. Simulation 4

The RMSE of the DOA estimates versus number of snapshots is presented in simulation 4. The direction of the incident wave is 117°, SNR = −12 dB, and the number of snapshots varies from 50 to 3050 with a step-size of 300. For demonstration, 100 times Monte Carlo are ran for each snapshot.

**Figure 5:**
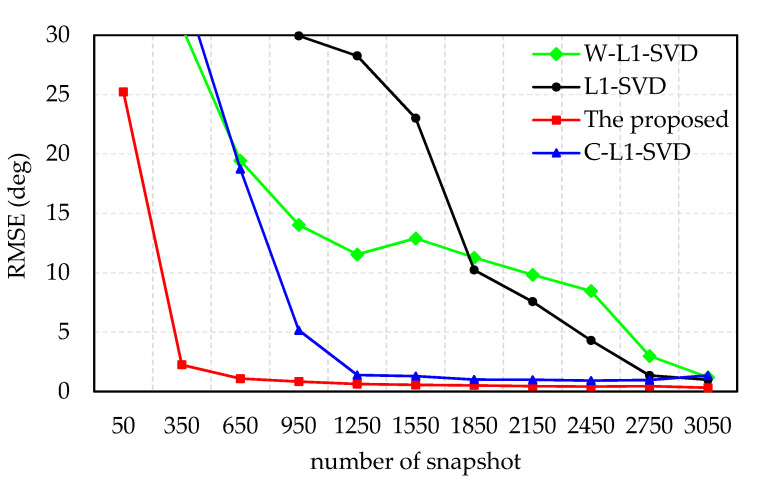
RMSE of the DOA estimates versus number of snapshots with −12 dB SNR.

Figure 5 indicates the RMSE of the DOA estimates versus number of snapshots with −12 dB SNR. When the SNR is the same, the RMSE of the DOA estimates decreases with the increase in the number of snapshots. The proposed algorithm is better than other algorithms in the RMSE of DOA estimation at low SNR, especially when the number of snapshots is small. The RMSE value of DOA estimation tends to be stable when the number of snapshots is beyond 350, while the numbers of the other three methods are: 1250 (C-L1-SVD), 2750 (L1-SVD), and 3050 (W-L1-SVD). Since the peak ratio of the weighted value of the proposed algorithm is greater than the other three algorithms, the l_1_-norm is closer to the l_0_-norm, and the main lobe of the spectrum is sharper and spurious peaks are suppressed. Therefore, the proposed algorithm can achieve a higher accuracy with fewer snapshots, which is conducive to saving calculation time.

#### 3.1.5. Simulation 5

As we known, the DOA estimation of the array signal requires prior knowledge of the number of signals, otherwise the estimation errors of the number of signals sometimes may lead to an error of the DOA estimation. Simulation 5 studies the relationship between the estimation of the number of signals and the DOA estimation at low SNR. The simulation conditions are set as follows: (1) The two OFDM signals have the same power, carrier frequency and bandwidth. (2) The directions of the two incident waves are 80° and 100°, respectively. (3) The number of snapshots is 3072. (4) SNR = −12 dB. (5) The number of signals is assumed to be *p* = 1, *p* = 2, and *p* = 4.

**Figure 6:**
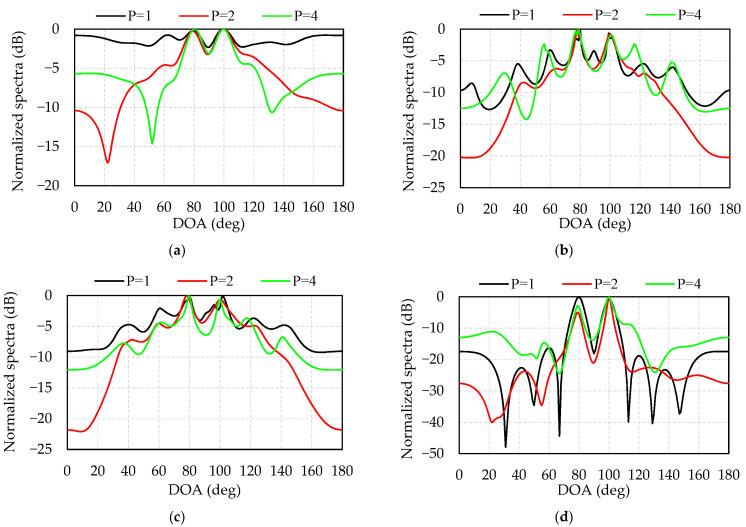
Sensitivity of the 4 algorithms to the assumed number of signals: (**a**) L1-SVD; (**b**)W-L1-SVD; (**c**) C-L1-SVD; (**d**) the proposed algorithm.

Figure 6 provides a comparison of the sensitivity of the four algorithms to the number of assumed signals. Figure 6a–d illustrates the normalized spectra of L1-SVD, W-L1-SVD, C-L1-SVD, and the proposed algorithms at *p* = 1, *p* = 2, and *p* = 4, respectively. Table 3 lists the estimated angles of these four algorithms. It can be seen from Table 4 and Figure 6 that when the number of signal estimation is accurate, the peak value of the spectrum is sharper and the side lobe is lower, leading to more accurate DOA estimation and better resolution. When the number of signals is underestimated, the number of weighted signal subspaces decreases, leading to an increase in the number and amplitude of side lobes. When the number of signals is overestimated, due to the noise subspace being added to the weights, the performance of the four algorithms becomes worse, especially for the W-L1-SVD algorithm. In comparison, the proposed algorithm is less sensitive to the misestimation of the number of signals. The peak ratio of the weighted value of the proposed algorithm is greater than that of the other three algorithms, which increases the sparsity of the solution, showing the merits of being more resistant to noise interference, and low sensitivity of misestimated signals.

### 3.2. Experiment Results and Analysis

**Figure 7:**
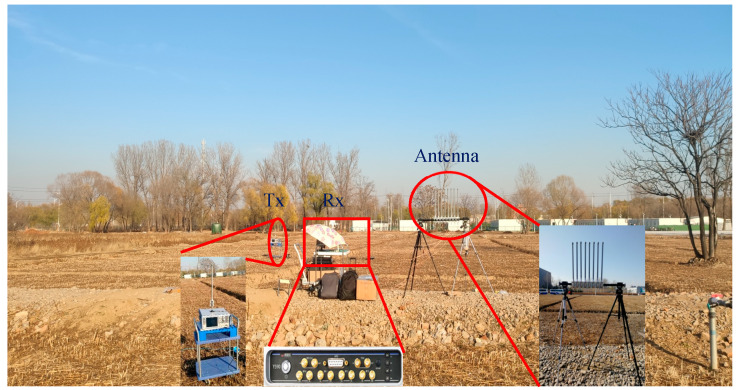
Photo taken during the measurement campaigns of the outdoor scenario pointing out the positions of the transmitter and the antenna array-based receiver.

In this section, we validate the proposed algorithm with an experiment operated in an outdoor scenario. Figure 7 is a photo taken during measurement at the northwest corner of Shahe campus of Beihang University. It depicts the experimental arrangement, in which an antenna array, a transmitter and a receiver are applied. The transmitter is placed 100 cm above the ground on the left, and the receiver is placed 150 cm above the ground on the right.

**Figure 8:**
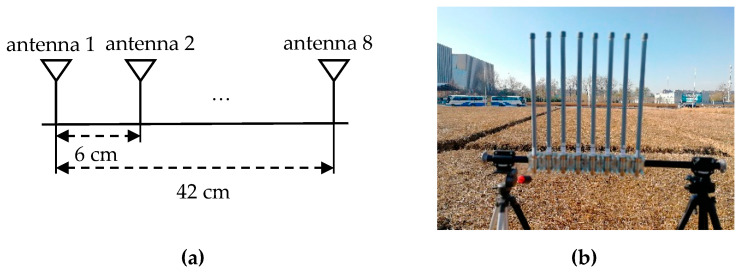
Eight-antenna uniform linear array: (**a**) schematic diagram; (**b**) physical picture.

A Rohde & Schwarz SMW200A vector signal generator was used as the transmitter, which can transmit an OFDM signal with a carrier frequency of 2.38 GHz, a bandwidth of 20 MHz, and output power of 35 dBm. Gaussian white noise with an SNR = 0 dB and SNR = 15 dB was added to the emission signal. Figure 8 displays the eight-antenna uniform linear array, Figure 8a is the schematic diagram, and Figure 8b is an eight-antenna uniform array made by us. A uniform linear array composed of eight fiberglass antennas, which are spaced 6 cm apart, was used to receive the incident signals. The receiver is a 4 × 4 multi-input multi-output software-defined radio platform, Weishirui Y590. In this experiment, an eight-channel synchronous receiver is constructed by two Y590s, whose IQ sampling rate is set as 32 MHz for signal acquisition. For experimental validation, two experiments are set out from different angles. The parameters of OFDM are the same as the simulation. An OFDM signal is incident to the antenna array from 117.2° and 90° directions, and the distances between the transmitter and the receiver are 21.9 and 19.5 m, which conform to the far-field conditions.

**Figure 9:**
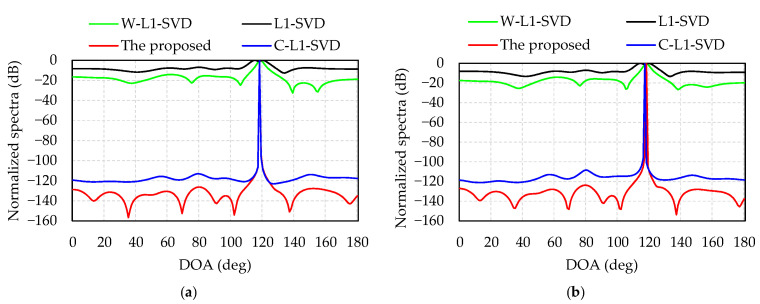
Normalized spectra during the measurement of outdoor scenario when the incident angle is 117.2°: (**a**) the SNR of emission signal is 0 dB; (**b**) the SNR of emission signal is 15 dB.

**Figure 10:**
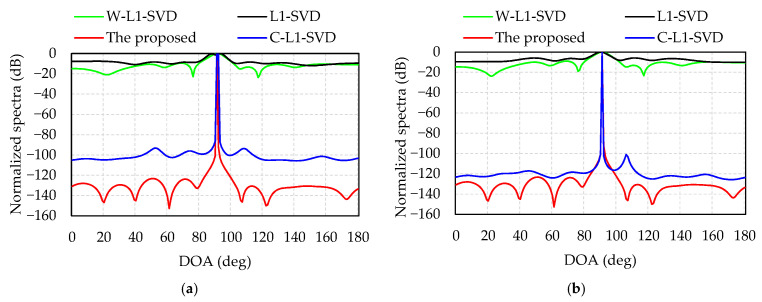
Normalized spectra during the measurement of outdoor scenario when the incident angle is 90°: (**a**) the SNR of emission signal is 0 dB; (**b**) the SNR of emission signal is 15 dB.

In total, 10,000 snapshots were used for data processing. Figure 9 presents the DOA estimation results of the four algorithms when the incident angle is 117.2°; Figure 9a,b shows the SNRs of emission signal are 0 and 15 dB. When the SNR = 0 dB, the DOA estimations of the proposed algorithm, L1-SVD, C-L1-SVD and W-L1-SVD are 118°, 116°, 118°, and 117°, respectively. When the SNR = 15 dB, the DOA estimations of the four algorithms are 118°, 115°, 117°, and 118°, respectively. Figure 10 shows the DOA estimation results of the four algorithms when the incident angle is 90°. Figure 10a illustrates the DOA estimations of algorithms are 91°, 93°, 92°, and 92° when the SNR of emission signal is 0 dB. Figure 10b shows that the DOA estimations of algorithms are 91°, 90°, 91°, and 91° when the SNR of emission signal is 15 dB. The DOA estimation error of the experimental results is a litter different from that of the simulations, which is caused by multi-path propagation and measurement error.

It can be seen from Figure 9 and Figure 10 that the proposed algorithm is able to accurately estimate the DOA. The DOA estimation performance (main lobe and side lobe) can be compared as follows: the proposed algorithm > C-L1-SVD > W-L1-SVD> L1-SVD. In the proposed algorithm, the main lobe is much sharper with lower side lobes compared with other algorithms. The experimental results demonstrate that the proposed algorithm determines the weighting matrix by optimizing the orthogonality of the subspace, thereby increasing the sparsity of the signal, resulting in a sharp main lobe and low side lobe of the spectrum, which can be used for DOA estimation of the UAV and pseudo base station. Therefore, the experiments verify the merits of the proposed algorithm.

## 4. Discussion

This paper proposes a DOA estimation method based on weighted l_1_-norm sparse representation. Compared with the traditional array signal processing method, the sparse representation DOA estimation algorithm requires fewer snapshots and fewer array elements. OFDM is often used in communication systems, such as WiFi, long term evolution (LTE), 5th generation mobile communication technology (5G) and unmanned aerial vehicle (UAV) video signals [20,21,22,23,24,25]; therefore, the DOA estimation of OFDM signal is discussed in this paper.

In the simulation, we compared the DOA estimation accuracy and resolution of the proposed algorithm with the other three algorithms at different SNRs. From simulations 1, 2, and 3, it can be concluded that the proposed algorithm can suppress the spurious peaks, with a sharp main lobe, which can improve the resolution and accuracy of DOA estimation. Simulation 4 shows that the proposed algorithm requires fewer snapshots at low SNR, which is beneficial to reduce calculation time. Simulation 5 shows that the proposed algorithm has low sensitivity of misestimated signals and good robustness. In the outdoor experiment, we designed an eight-antenna uniform linear array as the sensor and compared the DOA estimation accuracy at different SNRs of emission signal and different distances between the receiver and the transmitter. The advantages of the proposed algorithm are verified in the real-world scenarios.

The simulation results indicate that the proposed algorithm has a higher estimation accuracy and a better resolution with the lowest side lobes and the sharpest main lobes. Additionally, the proposed algorithm requires fewer snapshots and lower sensitivity of misestimated signals, especially at a low SNR. The experimental results show that the proposed algorithm is superior to other methods, with a narrower main lobe and lower side lobe. Considering that the long-distance DOA estimation is a challenge due to the low emission power of UAV video signal and other communication signals, the proposed algorithm has an engineering guiding significance for anti-UAV technology and the long-distance positioning of the pseudo base station.

## 5. Conclusions

In this paper, we present a sparse reconstruction of the DOA estimation algorithm based on weighted l_1_-norm. The weighted l_1_-norm is used as the minimum objective function to increase the signal sparsity, which is able to improve the accuracy of DOA estimation and suppress spurious peaks for the low SNR scenarios. Additionally, the OFDM signal of communication is taken as the simulated object. The simulated and experimental results show that the proposed algorithm has a sharper main lobe and lower side lobe, which can improve the resolution and estimate DOA accurately. Due to the above characteristics, the proposed algorithm also has an important guiding role in engineering, such as anti-UAV technology and pseudo base station positioning.

## Figures and Tables

**Table 1:** Simulation parameters of OFDM signal.

Carrier Frequency	Bandwidth	Number of the Subcarriers	Number of Effective Subcarriers	Subcarrier Spacing	Time of Useful Symbol Length	Number of Symbols
2038 MHz	20 MHz	256	192	78.125 KHz	12.8 us	10

**Table 2:** DOA estimates in Figure 4.

SNR (dB)	L1-SVD	W-L1-SVD	C-L1-SVD	The Proposed
−12	(79°, 100°)	(78°, 100°)	(78°, 100°)	(79°, 100°)
0	(80°, 100°)	(79°, 101°)	(79°, 101°)	(80°, 101°)

**Table 3:** The accuracy and average calculation time of the four algorithms over 100 times Monte Carlo.

Algorithm	RMSE (deg)	Average Calculation Time (s)
W-L1-SVD	3.57	2.993
L1-SVD	22.9	2.992
The proposed	0.98	2.997
C-L1-SVD	26.03	2.972

**Table 4:** DOA estimates in Figure 6.

Algorithm	*p* = 1	*p* = 2	*p* = 4
L1-SVD	(81°, 101°)	(80°, 101°)	(80°, 100°)
W-L1-SVD	(81°, 100°)	(79°, 101°)	(79°, 101°)
C-L1-SVD	(81°, 103°)	(79°, 101°)	(80°, 100°)
The proposed	(81°, 101°)	(80°, 101°)	(79°, 100°)

## Data Availability

Not applicable.
